# Cell-Based Phenotyping Reveals QTL for Membrane Potential Maintenance Associated with Hypoxia and Salinity Stress Tolerance in Barley

**DOI:** 10.3389/fpls.2017.01941

**Published:** 2017-11-16

**Authors:** Muhammad B. Gill, Fanrong Zeng, Lana Shabala, Guoping Zhang, Yun Fan, Sergey Shabala, Meixue Zhou

**Affiliations:** ^1^Department of Agronomy, College of Agriculture and Biotechnology, Zhejiang University, Hangzhou, China; ^2^School of Land and Food, University of Tasmania, Hobart, TAS, Australia

**Keywords:** H^+^-ATPase, *Hordeum vulgare*, hypoxia, membrane potential, salinity tolerance, waterlogging tolerance

## Abstract

Waterlogging and salinity are two major abiotic stresses that hamper crop production world-wide resulting in multibillion losses. Plant abiotic stress tolerance is conferred by many interrelated mechanisms. Amongst these, the cell’s ability to maintain membrane potential (MP) is considered to be amongst the most crucial traits, a positive relationship between the ability of plants to maintain highly negative MP and its tolerance to both salinity and waterlogging stress. However, no attempts have been made to identify quantitative trait loci (QTL) conferring this trait. In this study, the microelectrode MIFE technique was used to measure the plasma membrane potential of epidermal root cells of 150 double haploid (DH) lines of barley (*Hordeum vulgare* L.) from a cross between a Chinese landrace TX9425 and Japanese malting cultivar Naso Nijo under hypoxic conditions. A major QTL for the MP in the epidermal root cells in hypoxia-exposed plants was identified. This QTL was located on 2H, at a similar position to the QTL for waterlogging and salinity tolerance reported in previous studies. Further analysis confirmed that MP showed a significant contribution to both waterlogging and salinity tolerance. The fact that the QTL for MP was controlled by a single major QTL illustrates the power of the single-cell phenotyping approach and opens prospects for fine mapping this QTL and thus being more effective in marker assisted selection.

## Introduction

Waterlogging is one of the major abiotic stresses limiting agricultural production around the globe (Setter and Waters, 2003). It imposes several limitations on plants during their life span (Bailey-Serres and Voesenek, 2008; Shabala and Pottosin, 2014). Among them, the major constraint that a plant faces when exposed to waterlogging is either a complete unavailability or an inadequate supply of oxygen to submerged organs of flooding sensitive species (Armstrong and Drew, 2002). As a result, the transport of nutrients from roots to shoots is severely disturbed under waterlogged conditions (Smethurst et al., 2005; Colmer and Voesenek, 2009), which consequently affects plant growth and yield (Malik et al., 2001; Colmer et al., 2011). Salinity is another limiting factor for crop production. According to FAO (2008), almost 800 million hectares of global land area are affected by salinity which accounts for more than 20% of irrigated land area (Yamaguchi and Blumwald, 2005). Under saline conditions, excessive accumulation of Na^+^ and Cl^-^ results in their toxicity. Salinity stress also imposes osmotic and oxidative stress and interferes with the uptake and retention of other mineral elements such as K^+^ (Benito et al., 2014). Taken together, these factors lead to a disturbance of plant metabolism, reduced growth rates and plant yield. To meet the target of more than 70% increase in food production by 2050 (Garnett et al., 2013), it is important to improve the plant’s tolerance to cope with different abiotic stresses, including waterlogging and salinity.

Barley is considered to be a waterlogging sensitive (Zhou et al., 2012) and moderately salt tolerant cereal (Ullrich, 2002; Munns et al., 2006), although it shows significant variation over genotypes in waterlogging (Takeda and Fukuyama, 1986; Zhou, 2010) and salinity tolerance (Slavich et al., 1990; Jaradat et al., 2004). Many quantitative trait loci (QTLs) have been reported for waterlogging and salinity tolerance based on different physiological and agronomic traits. For waterlogging tolerance, QTL mapping was done targeting aerenchyma formation (Mano and Omori, 2009; Zhang et al., 2015), root porosity (Broughton et al., 2015), grain yield (Zaidi et al., 2015), leaf chlorosis (Li et al., 2008; Zhou et al., 2012; Ma et al., 2015) and plant biomass (Zhang et al., 2013) as the whole-plant based phenotypic traits. Several QTLs have also been identified for salinity tolerance by using many whole-plant based phenotypic indices such as shoot sodium content (Rivandi et al., 2010; Shavrukov et al., 2010), intercellular CO_2_ concentration (Liu et al., 2017), and germination rate (Mano and Komatsuda, 2002). However, none of these findings led to any major progress in creating stress-tolerant cultivars. Several reasons may explain this (Arzani and Ashraf, 2016). First, the statistical testing of null hypotheses (for example no QTL) is deeply embedded in the probability theory and conditions that create error variance lead to threats to statistical conclusion validity. The LOD threshold value for avoiding a false positive with a given confidence, say 95%, depends on the number of markers and the length of the genome. As a consequence, the literature describing QTL analyses might contain false-positive QTLs at too high a rate. The second major reason is the genotype (QTL) by environment interaction which often confounds with main effects of a QTL. This is specifically true to all field-based studies. Next, the quantitative genetic models are often based on certain (unrealistic) assumptions and also have strong background dependency.

From a physiological point of view, the major shortfall is that in nearly all cases the above phenotyping has been conducted at the whole-plant level, so each of the measured traits was conferred by multiple (and often unrelated) contributing mechanisms. As a result, multiple QTLs have been reported for each of these traits. For example 14 QTLs were associated with leaf chlorosis on chromosomes 1H, 2H, 3H, 4H, 5H, 6H, 7H for waterlogging tolerance (Li et al., 2008; Xu et al., 2012; Zhou et al., 2012) and 10 QTLs associated with plant height on chromosomes 1H, 2H, 4H, 5H, 7H for yield component (Li et al., 2005; Xue et al., 2010; Chutimanitsakun et al., 2011; Wang et al., 2015). The second reason is that very often the phenotypic indices used are not directly related to the mechanisms targeted and are, therefore, misleading. For example, measuring whole-shoot Na^+^ content (as in all studies; Genc et al., 2007; Haq et al., 2014; Tounsi et al., 2016) fails to account for differential ability of plants to sequester Na^+^ in leaf vacuoles; the trait considered to be the most crucial to confer salinity tissue tolerance. As a result, the amount of Na^+^ measured in the shoot will be the same for highly salt-sensitive species such as pea or rye and highly salt-tolerant halophyte species, but the impact on growth will be drastically different.

Thus, it appears that the real progress in plant breeding can be achieved only when plant phenotyping will directly target a contributing mechanism. This can be achieved only when such phenotyping is conducted at the cellular level.

Waterlogging and salinity tolerances are complex traits that are conferred by numerous physiological mechanisms (Jackson et al., 2009; Qiu et al., 2011; Shabala, 2011; Shabala et al., 2016). Amongst these, the plasma membrane (PM) H^+^-ATPases play a central role in cell ionic homeostasis and stress signaling and adaptation. Channel-mediated root nutrient acquisition depends on the electric potential difference [membrane potential (MP)] across the PM, which is controlled by the H^+^-ATPase activity (Palmgren and Nissen, 2011). H^+^ pumps also create a proton motive force for the secondary active ion transport (Shabala et al., 2016). The strong correlation between root PM H^+^-ATPase activity and an overall salinity stress tolerance was found in many species (Chen et al., 2007; Bose et al., 2014; Lei et al., 2014). The same is true for waterlogging stress. Most of the membrane transporters are voltage gated in nature, and the PM is significantly depolarized (typically by 40 to 70 mV) under oxygen-limited conditions due to insufficient ATP availability (Teakle et al., 2013; Zeng et al., 2014).

H^+^-ATPase-mediated maintenance of a highly negative MP is one of the key elements of maintenance of intracellular K^+^ homeostasis. Potassium (K^+^) is an essential and most abundant nutrient which plays significant roles in plant growth. K^+^ is involved in the cell turgor pressure maintenance, cell elongation, stress signaling, and osmoregulation (Dreyer and Uozumi, 2011; Shabala and Pottosin, 2014). Stress-induced membrane depolarization activates outward-rectifying K^+^ efflux channels (GORK in *Arabidopsis*), resulting in a massive K^+^ loss under both hypoxia (Elzenga and van Veen, 2010) and salinity stress conditions (Chen et al., 2005), and leading to a significant reduction in plant K^+^ content (Smethurst et al., 2005; Board, 2008). This decline in K^+^ content results in severe yield penalties (Drew and Sisworo, 1979; Robertson et al., 2009) and, in extreme cases, in the loss of the cell viability (Shabala et al., 2016). The plant’s ability to survive under waterlogged and saline conditions could be improved by improving its K^+^ retention capacity (Wang et al., 2013). Interestingly, plants often respond to salinity stress by an increase in the GORK transcript level (Adem et al., 2014; Chakraborty et al., 2016) suggesting that it is a post-translational regulation of GORK channel that is crucial for adaptive responses to stress. As mentioned above, voltage gating is arguably the most essential factor in this regulation. Thus, finding the QTL responsible for such gating may open a novel and previously unexplored avenue for improving abiotic (salinity and waterlogging) stress tolerance via enhanced K^+^ retention.

In this study, we have adopted a new method to phenotype plants at the single-cell level, to account for the tissue-specific expression of transporters, and identify a QTL responsible for the maintenance of negative MP under hypoxic conditions. This method relied on using the microelectrode MIFE technique, and has been applied to screen 150 barley double haploid (DH) lines from a cross between TX9425 and Naso Nijo under hypoxia (waterlogging). Analyses were conducted to identify the linkage between this trait and waterlogging/salinity tolerances. For the first time in the literature, we report a major QTL for the MP. This finding may open new avenues for future breeding programs to develop more tolerant varieties.

## Materials and Methods

### Plant Material

A total of 150 DH lines from a cross between TX9425 and Naso Nijo (Xu et al., 2012) were used in this study for MP measurements. TX9425 is Chinese, two-rowed barley variety which is tolerant to waterlogging and salinity (Pang et al., 2004; Zhou et al., 2007) and shows a few exceptional agronomic characteristics (Wang et al., 2010) and resistance to some diseases (Li et al., 2009; Li and Zhou, 2011). Naso Nijo is a Japanese malting barley variety with good agronomic characteristics but is sensitive to both waterlogging (Pang et al., 2004) and salinity (Xu et al., 2012).

Seeds were surface sterilized with 10% commercial bleach (NaClO 42 g L^-1^; Pental Products, Shepparton, VIC, Australia), thoroughly rinsed with tap water for at least 30 min and then grown in wet paper rolls with basic salt media (BSM) solution (0.5 mM KCl + 0.1 mM CaCl_2_, pH 5.6) in the dark for 3 days at room temperature (25 ± 1°C). Two treatments were used in the present experiment: (1) control (BSM, aerated); and (2) hypoxia (BSM solution made with 0.2% agar and bubbled with N_2_ gas). For the treatment with agar, the stagnant solution was prepared by adding agar (Cat. No. LP0011, Oxoid, Hampshire, United Kingdom) to the BSM solution at a ratio of 0.2% (w/v) and boiled, then cooled overnight to room temperature with magnetic stirring to prevent lump formation. The agar solutions were pre-bubbled with high purity N_2_ (Cat. No. 032G, BOC Gases, Hobart, TAS, Australia) for at least 1 h before being used in the experiment.

### Evaluation of the DH Lines for Waterlogging and Salinity Tolerance

All the details related to waterlogging and salt tolerance evaluation experiments are given in our previous publication (Xu et al., 2012). In brief, for waterlogging tolerance evaluation DH lines generated from a cross between TX9425 and Naso Nijo were subjected to waterlogging for 9 weeks until sensitive lines died. A collective scoring system was used, with scoring index 0 indicating no damage and index 10 given to dead plants. Plants with scores 0–5 showed various levels of chlorosis and those with scores 6 or above showing a substantial percentage of necrotic leaves, under waterlogged conditions.

To evaluate salt tolerance, seeds of the DH lines were sown in 40-L containers filled with a pine bark/loam-based potting mix with premixed slow release fertilizer. After germination (7 days after sowing) 200 mM NaCl treatment was applied and maintained until data collected. Salt tolerance was assessed by combining scores for leaf chlorosis and plant survival after 7 weeks of sowing and conducted on a similar way to the one described above for waterlogging.

### Membrane Potential Measurements

Membrane potential values were measured from root epidermal cells of intact barley seedlings. Conventional microelectrodes (Harvard Apparatus) were filled with 1 M KCL and connected to MIFE electrometer via Ag/AgCl half-cell. During MP measurement, the microelectrode with a tip diameter of 0.5 μm was manually impaled into the epidermal cells of mature root zone (5 mm from shoot base) using a functioned 3D-micromanipulator (MHW-4, Narishige, Tokyo, Japan). MP values were recorded by the MIFE CHART software for at least 2 min after stabilization (Newman, 2001).

**FIGURE 1:**
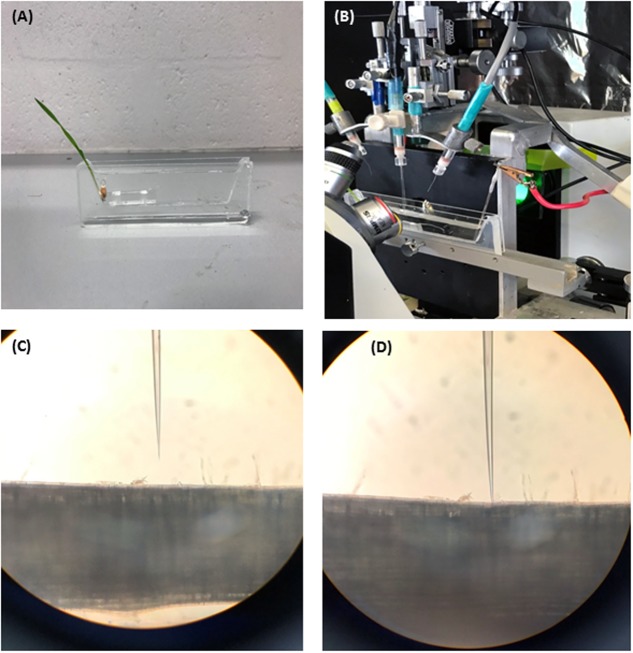
Four steps of experimental procedure are illustrated. **(A)** Seedling is imbobilized in a vertical chamber and treated with hypoxia solution (N_2_ bubbled 0.2% agar). **(B)** The vertical chamber is mounted in faraday cage for membrane potential measurements. **(C)** Electrode is positioned next to root epidermis. **(D)** Electrode is impaled into the root cell for membrane potential measurements.

Prior to measurement a 3-day old seedling was taken from a paper roll and mounted in a vertical chamber and treated then with hypoxia solution. The measuring chamber was filled with hypoxia solution with the coleoptile being above the surface of the solution. Roots were kept under stagnant conditions for 48 h. The seedlings were then placed into the Faraday cage for MP measurements. For each DH line, MP values were measured from roots of 5–6 individual seedlings after 48 h of treatment. At least four measurements were taken from each seedling. The overview of the experimental procedure is further illustrated in**Figure 1**.

### Map Construction and QTL Analysis

Genomic DNA of the DH population was extracted from the leaf tissue of 4-week old seedlings. A total of 28047 DArT and 8928 SNP markers were used for genotyping. After removing markers with greater distortion and missing data, 4788 markers were chosen for map construction. A new genetic map of the DH population was constructed using the software package JoinMap 4.0 (Van Ooijen, 2006). QTL analysis was conducted using the software package MapQTL 6.0 (Van Ooijen, 2009). Interval mapping (IM) was firstly used to detect the major QTL. The nearest marker at the QTL from IM was chosen as a cofactor in the multiple QTL model (MQM). Logarithm of the odds (LOD) threshold values applied to declare the presence of a QTL were estimated by performing the genome wide permutation tests implemented in MapQTL version 6.0 using at least 1000 permutations of the original data set for each trait, resulting in a 95% LOD threshold around 3.0. To determine the effects of physiological traits on waterlogging and salinity tolerance, QTL for both waterlogging and salinity tolerance were re-analyzed by using various physiological traits as covariates. Maps showing the QTL position and LOD values were generated using MAPCHART (Voorrips, 2002).

## Results

### Membrane Potential Values of Parents and DH Lines under Hypoxia Stress

**FIGURE 2:**
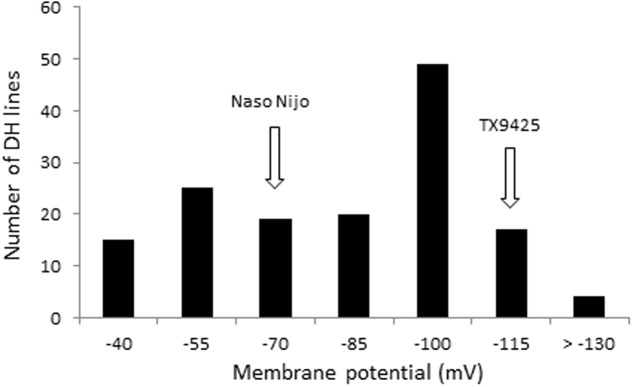
The frequency distribution for membrane potential (MP) under hypoxia (0.2% agar) stress of DH lines derived from a cross of TX9425 and Naso Nijo.

The protocol for MP measurements from barley roots via microelectrode MIFE technique is shown in **Figure 1**. Both parent cultivars showed a significant difference in MP values when measured from epidermal root cells of barley after 48 h of hypoxia stress. Under hypoxia stress, MP values of the waterlogging tolerant parent TX9425 were significantly more negative (-125.3 ± 3.3 mV) than of sensitive parent Naso Nijo (-83.4 ± 2.9 mV) (**Table 1**). The DH lines from the cross between TX9425 and Naso Nijo also showed significant difference in values of MP when exposed to hypoxia for 48 h. **Figure 2** shows the frequency distribution of waterlogging tolerance based on the MP values under hypoxia stress. A continues distribution was found for MP with values ranging from -41 to -138 mV (**Table 1**). Analysis of variance (ANOVA) for MP showed a significant difference (*P* < 0.001) between DH lines under hypoxia stress (**Supplementary Table S1**).

**Table 1:** Effects of hypoxia (N_2_ bubbled 0.2% agar) stress on membrane potential values of parents and DH lines.

Cultivars	Membrane potential (mV)
TX9425	-125.33 ± 7.34
Naso Nijo	-85.42 ± 6.96
DH lines average	-91.17 ± 14.54
DH lines range	-40.97 ±-137.52


*Data are mean values ± SE.*

### QTL for Membrane Potential

**FIGURE 3:**
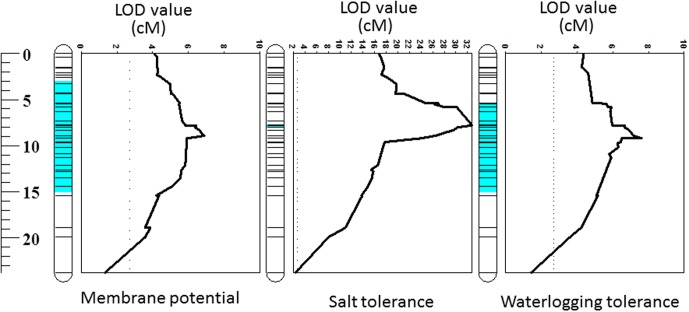
Quantitative trait loci (QTL) for membrane potential, salt and waterlogging tolerances on 2HS. The figures related to salt and waterlogging tolerance incorporate data published by Xu et al. (2012). The full length of chromosome 2H is also displayed in this study.

One major QTL for MP was identified on chromosome 2H which was designated as *QMP.TxNn.2H*. This QTL was detected close to 8613801D2 marker at the position of 8.85 cM and explained 22% of the phenotypic variation (**Table 2**). The position of the QTL identified in this study was the same as that for waterlogging tolerance (Xu et al., 2012) (**Figure 3**).

**Table 2:** Quantitative trait loci (QTL) on 2HS for membrane potential, salt and waterlogging tolerance detected in the DH population of TX9425 × Naso Nijo.

Traits	Linkage group	Nearest marker	Position (cM)	LOD	R^2^ (%)	Co-variate
MP	2H	8613801D2	8.85	6.89	22.0	
	2H	8613801D2	8.85	6.2	19.5	Waterlogging
	2H	8613801D2	8.85	1.99	5.7	Salt
Waterlogging	2H	3258828D2	9.21	7.61	21	
	2H	3258828D2	9.21	5.83	18.4	MP
Salt	2H	3259260S2	7.79	32.79	63.7	
	2H	3259260S2	7.79	26.29	50.8	MP


### Contribution of Membrane Potential to Waterlogging and Salt Tolerance

**FIGURE 4:**
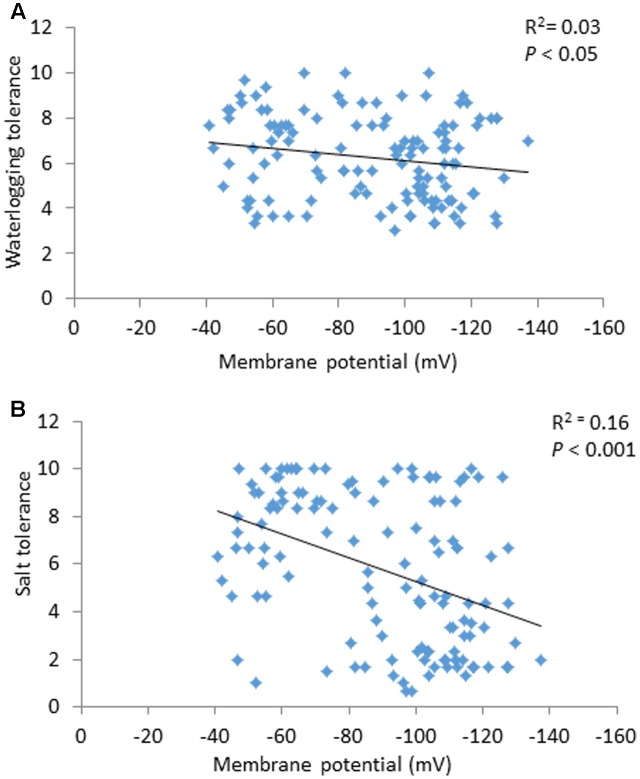
Correlation between membrane potential and waterlogging tolerance scores **(A)** and between membrane potential and salt tolerance scores **(B)**.

**FIGURE 5:**
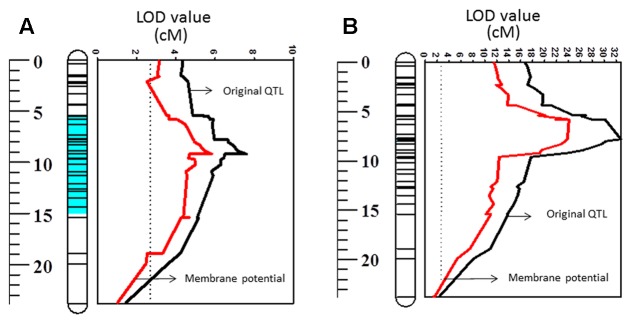
Quantitative trait loci associated with waterlogging tolerance (LOD values) on 2HS **(A)** and QTL associated with salt tolerance (LOD values) on 2HS **(B)**. Black line: LOD value of original QTL; Red line: LOD value of QTL when membrane potential is used as a covariate.

Membrane potential showed a significant (*P* < 0.05) correlation with waterlogging tolerance (**Figure 4A**). This is further confirmed by QTL analysis for waterlogging tolerance using MP as a covariate. As shown in **Figure 5A**, the LOD value of the QTL on 2H for waterlogging tolerance showed a slight reduction when MP was used as a covariate. The percentage of the phenotypic variation (R^2^) determined by the QTL also showed a slight reduction, from 21.0 to 18.4% (**Table 2**). MP also showed a close and significant correlation (*P* < 0.001) with salt tolerance (**Figure 4B**). Correlation between MP and salt tolerance is higher than the correlation between MP and waterlogging tolerance. When MP was used as covariate, LOD value and R^2^ of the QTL for salt tolerance reduced from 32.8 to 26.3 and 63.7 to 50.8, respectively (**Figure 5B** and **Table 2**).

### QTL for MP When Using Waterlogging and Salt Tolerance As Covariates

**FIGURE 6:**
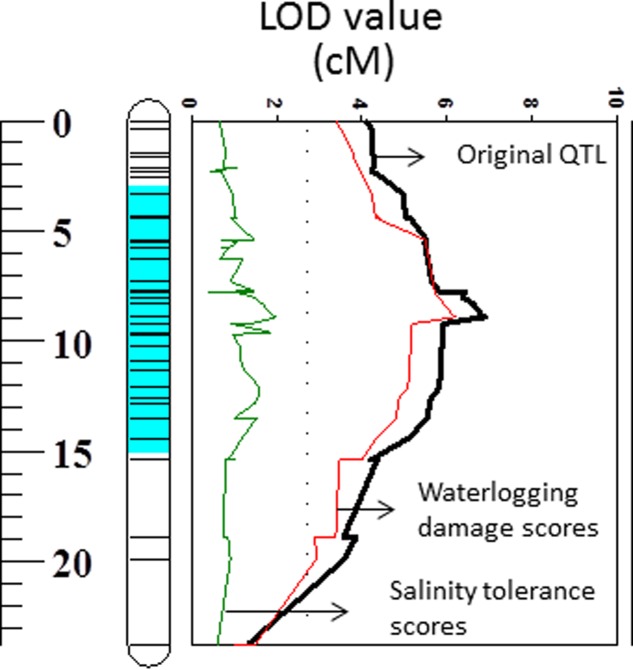
Quantitative trait loci associated with membrane potential (LOD values) on 2HS. Black line: LOD value of original QTL; Red line: LOD value of QTL when waterlogging damage scores are used as a covariate; Green line: LOD value of QTL when salinity tolerance scores are used as a covariate.

The weak correlation with waterlogging tolerance and strong correlation with salt tolerance were further confirmed by reverse QTL analysis, i.e., analysis of QTL for MP using either waterlogging or salt tolerance as a covariate. When such analysis was conducted by using waterlogging damage scores as a covariate, only slight reductions in both LOD and R^2^ of the QTL for MP were found while the QTL for MP became insignificant when salt tolerance scores were used as covariates (**Figure 6**).

## Discussion

Tolerance to abiotic stresses is an important breeding objective. Great efforts have been made to identify mechanisms conferring waterlogging/salinity tolerance and finding QTL for the tolerance using different screening systems (Aslam et al., 1993; Mano and Takeda, 1997; Foolad et al., 2001; Lee et al., 2006, 2007; Pang et al., 2007; Chen H. et al., 2008; Chen Z. et al., 2008; Farshadfar et al., 2008; Xue et al., 2009; Fan et al., 2015). However, the practical outcomes are still disappointingly small. Both waterlogging and salinity tolerances are highly complicated traits that are controlled by many different mechanisms. Direct selection of the overall tolerance is very hard thus breeders rely on molecular markers linked to the tolerance. Most QTL identified for waterlogging/salinity tolerance are based on plant survival rate, plant healthiness and leaf chlorosis under stress (Li et al., 2008; Xue et al., 2010; Xu et al., 2012; Zhou et al., 2012; Ma et al., 2015; Zhang et al., 2016). While these traits are convenient for high throughput screening, they are not directly related to the mechanisms conferring the tolerance. As a result, fine mapping of these QTLs to provide reliable markers to breeders is very difficult, even if possible in principle due to the very large number of QTLs involved.

Much more promising is an approach when specific QTLs are linked directly with appropriate mechanisms. Since most of the mechanisms are expected to be controlled by just one or two QTLs, these are much easier to fine map. A good example of this success is for barley waterlogging tolerance, the major QTL for waterlogging tolerance on 4H (Li et al., 2008; Zhou, 2011; Zhou et al., 2012) is due to the formation of aerenchyma under stress which is controlled by a single major QTL (Broughton et al., 2015; Zhang et al., 2016, 2017) and the gene has been fine mapped to a <2 cM region with closely linked markers being available for breeders to use.

The PM is responsible for the maintenance of ionic and electric gradients between the cytosol and external media and thus essential for intracellular ionic homeostasis. It is also an important component of the signal transduction in plants under stress conditions (Kim et al., 2007). PM depolarization is one of the common features between salinity and waterlogging stresses, leading to a substantial disruption in the ionic homeostasis (Palmgren and Nissen, 2011; Shabala et al., 2016) which contributes to metabolic disturbances and ultimately determines the cell’s fate. The electrogenic H^+^-ATPase pumps plays a significant role in maintaining negative potential of the PM. Oxygen limited conditions resulted in a significant depolarization of the PM due to huge decline in ATP availability to fuel H^+^-ATPase. The PM is also depolarized as a result of massive Na^+^ uptake under saline conditions. In our experiment, waterlogging/salt tolerant variety, TX9425, showed a much better ability to maintain MP under hypoxia stress than waterlogging/salt sensitive variety, Naso Nijo (**Table 1**). The DH population from these two varieties showed a wide range of segregation (**Figure 2**) and a major QTL (*QMP.TxNn.2H*) for MP (**Figure 3**) was identified. This QTL is located on the short arm of chromosome 2H and explained 22% of phenotypic variation (**Table 2**). The fact that only one single major QTL was identified in this population makes it easier to further fine map the gene.

A large number of QTLs for different stress tolerances were reported at this position (Zhang et al., 2017), which include waterlogging (Zhou, 2011; Xu et al., 2012), salinity (Xu et al., 2012), and drought (Fan et al., 2015) with some being identified from the same DH population used in this study. Importantly, all these stresses are known to affect H^+^-ATPase activity and depolarize the PM (Shabala et al., 2016). *Arabidopsis* mutants lacking an H^+^-ATPase isoform showed increased sensitivity to salt and accumulate higher concentrations of Na^+^ in leaves compared to wild type plants (Vitart et al., 2001). On the contrary, expressing *Arabidopsis thaliana* V-ATPase subunit C in barley improved plant performance under saline condition by enabling better osmotic adjustment (Adem et al., 2017). When comparing MP with waterlogging/salt tolerance scores from the same population, MP showed significant correlations with both waterlogging and salinity tolerance (**Figure 4**). Further QTL mapping of different traits was conducted using other related traits as covariates, which has been proved to be effective in confirming the relationship between different traits (Fan et al., 2015). When MP was used as a covariate the LOD value and R^2^ of the QTL on 2H for both waterlogging and salt tolerances reduced with less effect on waterlogging tolerance, confirming the weak linkage between MP and waterlogging tolerance and greater contribution of MP to salt tolerance (**Figure 5** and **Table 2**). The reason for the different contribution of MP to the different stresses may be related to the difference in the principal causes for membrane depolarization upon low oxygen and salinity (e.g., compromised mitochondrial operation for the former, and massive influx of Na^+^ for the latter).

It is not clear at this stage what specific gene contributes to the better maintenance of higher MP values in hypoxia-affected barley roots. The nearest marker of the QTL detected in this study was located around 8.28 cM, and the list of all candidate genes within 5 cM distance to 8.28 cM is given in **Supplementary Table S2**. No known subunits of H^+^-ATPase appear to be present in the vicinity of the reported marker suggesting that it was a *regulation* rather than the physical presence of the H^+^-ATPase protein matter for MP maintenance. It was previously shown that the plant’s ability to maintain negative MP was not attributed to changes in H^+^-ATPase transcripts or the actual amount of protein (reviewed in Shabala et al., 2016) but rather regulated by the post-translational modifications. This regulation may occur via multiple pathways; one of them involves hormonal signaling. For example, it is known that ABA dephosphorylates the penultimate Thr residue on the H^+^-ATPase, resulting in deactivation of the pump (Hayashi et al., 2014), and the protein kinase PKS5/CIPK11, an important element of ABA signaling cascade (Lumba et al., 2014), reducing the activity of the H^+^-ATPase (Fuglsang et al., 2007). Additionally, an overexpression of a tomato 14-3-3 homolog (GRF9) resulted in an increased H^+^-ATPase activity (Hu et al., 2015). The above annotated contigs for the 3 to 13 cM region on chromosome 2H contains a large number of kinases and kinase-like proteins. Also, two genes related to energy metabolism were detected in the vicinity of the marker. These were ectonucleoside triphosphate diphosphohydrolase 5 (E-NTPDase) and dihydrolipoyllysine-residue succinyltransferase component of 2-oxoglutarate dehydrogenase complex. E-NTPDases break down nucleoside tri- and diphosphates to nucleoside monophosphates and inorganic phosphate (Pi) and perform a wide range of functions. This includes purinergic signaling and control of the ATO concentration in ER and Goldgi lumen to regulate ATP-dependent processes (Massalski et al., 2015). It remains therefore a task for a future studies to answer the question which of them is responsible for regulation of H^+^-ATPase activity under stress conditions.

## Conclusion

A major QTL for MP maintenance under hypoxia was identified using cell-based phenotyping involving microelectrode MIFE technique. The QTL is located at a similar position to that for waterlogging and salinity tolerance on chromosome 2H. MP showed a weak but significant linkage with waterlogging tolerance and a strong linkage with salt tolerance. As only one single major QTL was responsible for MP, this makes it easier to fine map this QTL and effectively use this gene in pyramiding different tolerance mechanisms in breeding programs.

## Author Contributions

MG conducted phenotyping, data analysis and prepared the draft; YF assisted mapping, QTL analysis and candidate gene search; FZ, LS, and GZ helped in manuscript preparation and revision; SS and MZ conceived and supervised the project, reviewed and revised the manuscript.

## Conflict of Interest Statement

The authors declare that the research was conducted in the absence of any commercial or financial relationships that could be construed as a potential conflict of interest.

## Supplementary Material

**TABLE S1 |** ANOVA of membrane potential values under waterlogging (hypoxia) stress.

**TABLE S2 |** List of annotated proteins search by contig blast from the website (http://webblast.ipk-gatersleben.de/barley/).

## References

[B1] Adem G. D. Roy S. Huang Y. Chen Z. Wang F. Zhou M. (2017). Expressing *Arabidopsis thaliana* V-ATPase subunit C in barley (*Hordeum vulgare* L.) improves plant performance under saline condition by enabling better osmotic adjustment. *Funct. Plant Biol.* 10.1071/FP17049 32480640

[B2] Adem G. D. Roy S. J. Zhou M. Bowman J. P. Shabala S. (2014). Evaluating contribution of ionic, osmotic and oxidative stress components towards salinity tolerance in barley. *BMC Plant Biol.* 14:113. 10.1186/1471-2229-14-113 24774965 PMC4021550

[B3] Armstrong W. Drew M. (2002). “Root growth and metabolism under oxygen deficiency,” in *Plant Roots: The Hidden Half*, eds Wasel Y. eshel A. kafkafi U. (New York, NY: Marcel Dekker, Inc.), 729–761.

[B4] Arzani A. Ashraf M. (2016). Smart engineering of genetic resources for enhanced salinity tolerance in crop plants. *Crit. Rev. Plant Sci.* 35 146–189. 10.1080/07352689.2016.1245056

[B5] Aslam M. Qureshi R. Ahmed N. (1993). A rapid screening technique for salt tolerance in rice (*Oryza sativa* L.). *Plant Soil* 150 99–107. 10.1007/BF00779180

[B6] Bailey-Serres J. Voesenek L. (2008). Flooding stress: acclimations and genetic diversity. *Annu. Rev. Plant Biol.* 59 313–339. 10.1146/annurev.arplant.59.032607.092752 18444902

[B7] Benito B. Haro R. Amtmann A. Cuin T. A. Dreyer I. (2014). The twins K^+^ and Na^+^ in plants. *J. Plant Physiol.* 171 723–731. 10.1016/j.jplph.2013.10.014 24810769

[B8] Board J. (2008). Waterlogging effects on plant nutrient concentrations in soybean. *J. Plant Nutr.* 31 828–838. 10.1080/01904160802043122 29091286

[B9] Bose J. Rodrigo-Moreno A. Lai D. Xie Y. Shen W. Shabala S. (2014). Rapid regulation of the plasma membrane H^+^-ATPase activity is essential to salinity tolerance in two halophyte species, *Atriplex lentiformis* and *Chenopodium quinoa*. *Ann. Bot.* 115 481–494. 10.1093/aob/mcu219 25471095 PMC4332608

[B10] Broughton S. Zhou G. Teakle N. L. Matsuda R. Zhou M. O’Leary R. A. (2015). Waterlogging tolerance is associated with root porosity in barley (*Hordeum vulgare* L.). *Mol. Breed.* 35 1–15. 10.1007/s11032-015-0243-3

[B11] Chakraborty K. Bose J. Shabala L. Shabala S. (2016). Difference in root K^+^ retention ability and reduced sensitivity of K^+^-permeable channels to reactive oxygen species confer differential salt tolerance in three Brassica species. *J. Exp. Bot.* 67 4611–4625. 10.1093/jxb/erw236 27340231 PMC4973732

[B12] Chen H. Cui S. Fu S. Gai J. Yu D. (2008). Identification of quantitative trait loci associated with salt tolerance during seedling growth in soybean (*Glycine max* L.). *Aus. J. Agri. Res.* 59 1086–1091. 10.1186/1471-2229-13-161 24134188 PMC4015884

[B13] Chen Z. Pottosin I. I. Cuin T. A. Fuglsang A. T. Tester M. Jha D. (2007). Root plasma membrane transporters controlling K^+^/Na^+^ homeostasis in salt-stressed barley. *Plant Physiol.* 145 1714–1725. 10.1104/pp.107.110262 17965172 PMC2151677

[B14] Chen Z. Shabala S. Mendham N. Newman I. Zhang G. Zhou M. (2008). Combining ability of salinity tolerance on the basis of NaCl- induced K^+^ flux from roots of barley. *Crop Sci.* 48 1382–1388. 10.2135/cropsci2007.10.0557

[B15] Chen Z. H. Newman I. Zhou M. X. Mendham N. Zhang G. P. Shabala S. (2005). Screening plants for salt tolerance by measuring K^+^ flux: a case study for barley. *Plant Cell Environ.* 28 1230–1246. 10.1111/j.1365-3040.2005.01364.x

[B16] Chutimanitsakun Y. Nipper R. W. Cuesta-Marcos A. Cistué L. Corey A. Filichkina T. (2011). Construction and application for QTL analysis of a restriction site associated DNA (RAD) linkage map in barley. *BMC Genomics* 12:4. 10.1186/1471-2164-12-4 21205322 PMC3023751

[B17] Colmer T. Voesenek L. (2009). Flooding tolerance: suites of plant traits in variable environments. *Funct. Plant Biol.* 36 665–681. 10.1071/FP09144 32688679

[B18] Colmer T. D. Winkel A. Pedersen O. (2011). A perspective on underwater photosynthesis in submerged terrestrial wetland plants. *AoB Plants* 2011:plr030. 10.1093/aobpla/plr030 22476500 PMC3249690

[B19] Drew M. Sisworo E. (1979). The development of waterlogging damage in young barley plants in relation to plant nutrient status and changes in soil properties. *New Phytol.* 82 301–314. 10.1111/j.1469-8137.1979.tb02656.x

[B20] Dreyer I. Uozumi N. (2011). Potassium channels in plant cells. *FEBS J.* 278 4293–4303. 10.1111/j.1742-4658.2011.08371.x 21955642

[B21] Elzenga J. T. M. van Veen H. (2010). “Waterlogging and plant nutrient uptake,” in *Waterlogging Signalling and Tolerance in Plants*, (Berlin: Springer), 23–35. 10.1007/978-3-642-10305-6_2

[B22] Fan Y. Shabala S. Ma Y. Xu R. Zhou M. (2015). Using QTL mapping to investigate the relationships between abiotic stress tolerance (drought and salinity) and agronomic and physiological traits. *BMC Genomics* 16:43. 10.1186/s12864-015-1243-8 25651931 PMC4320823

[B23] FAO (2008). *FAO Land and Plant Nutrition Management Service*. Available at: http://www.fao.org/ag/agl/agll/spush

[B24] Farshadfar E. Safavi S. Aghaee-Sarbarzeh M. (2008). Locating QTLs controlling salt tolerance in barley using wheat-barley disomic addition lines. *Asian J. Plant Sci.* 7 149–155. 10.3923/ajps.2008.149.155

[B25] Foolad M. Zhang L. Lin G. (2001). Identification and validation of QTLs for salt tolerance during vegetative growth in tomato by selective genotyping. *Genome* 44 444–454. 10.1139/g01-030 11444704

[B26] Fuglsang A. T. Guo Y. Cuin T. A. Qiu Q. Song C. Kristiansen K. A. (2007). *Arabidopsis* protein kinase PKS5 inhibits the plasma membrane H^+^-ATPase by preventing interaction with 14-3-3 protein. *Plant Cell* 19 1617–1634. 10.1105/tpc.105.035626 17483306 PMC1913743

[B27] Garnett T. Appleby M. C. Balmford A. Bateman I. J. Benton T. G. Bloomer P. (2013). Sustainable intensification in agriculture: premises and policies. *Science* 341 33–34. 10.1126/science.1234485 23828927

[B28] Genc Y. Mcdonald G. K. Tester M. (2007). Reassessment of tissue Na^+^ concentration as a criterion for salinity tolerance in bread wheat. *Plant Cell Environ.* 30 1486–1498. 10.1111/j.1365-3040.2007.01726.x 17897418

[B29] Haq T. U. Akhtar J. Steele K. A. Munns R. Gorham J. (2014). Reliability of ion accumulation and growth components for selecting salt tolerant lines in large populations of rice. *Funct. Plant Biol.* 41 379–390. 10.1071/FP13158 32480998

[B30] Hayashi Y. Takahashi K. Inoue S.-I. Kinoshita T. (2014). Abscisic acid suppresses hypocotyl elongation by dephosphorylating plasma membrane H^+^-ATPase in *Arabidopsis thaliana*. *Plant Cell Physiol.* 55 845–853. 10.1093/pcp/pcu028 24492258

[B31] Hu D.-G. Sun M.-H. Sun C.-H. Liu X. Zhang Q.-Y. Zhao J. (2015). Conserved vacuolar H^+^-ATPase subunit B1 improves salt stress tolerance in apple calli and tomato plants. *Sci. Hortic.* 197 107–116. 10.1016/j.scienta.2015.09.019

[B32] Jackson M. B. Ishizawa K. Ito O. (2009). Evolution and mechanisms of plant tolerance to flooding stress. *Ann. Bot.* 103 137–142. 10.1093/aob/mcn242 19145714 PMC2707321

[B33] Jaradat A. Shahid M. Al-Maskri A. (2004). Genetic diversity in the batini barley landrace from Oman. *Crop Sci.* 44 997–1007. 10.2135/cropsci2004.9970

[B34] Kim B. G. Waadt R. Cheong Y. H. Pandey G. K. Dominguez-Solis J. R. Schültke S. (2007). The calcium sensor CBL10 mediates salt tolerance by regulating ion homeostasis in *Arabidopsis*. *Plant J.* 52 473–484. 10.1111/j.1365-313X.2007.03249.x 17825054

[B35] Lee S. Y. Ahn J. H. Cha Y. S. Yun D. W. Lee M. C. Ko J. C. (2006). Mapping of quantitative trait loci for salt tolerance at the seedling stage in rice. *Mol. Cells* 21 192–196. 16682812

[B36] Lee S. Y. Ahn J. H. Cha Y. S. Yun D. W. Lee M. C. Ko J. C. (2007). Mapping QTLs related to salinity tolerance of rice at the young seedling stage. *Plant Breed.* 126 43–46. 10.1111/j.1439-0523.2007.01265.x

[B37] Lei B. Huang Y. Sun J. Xie J. Niu M. Liu Z. (2014). Scanning ion-selective electrode technique and X-ray microanalysis provide direct evidence of contrasting Na^+^ transport ability from root to shoot in salt-sensitive cucumber and salt-tolerant pumpkin under NaCl stress. *Physiol. Plant.* 152 738–748. 10.1111/ppl.12223 24813633

[B38] Li H. Zhou M. (2011). Quantitative trait loci controlling barley powdery mildew and scald resistances in two different barley doubled haploid populations. *Mol. Breed.* 27 479–490. 10.1007/s11032-010-9445-x

[B39] Li H. Zhou M. Liu C. (2009). A major QTL conferring crown rot resistance in barley and its association with plant height. *Theor. Appl. Genet.* 118 903–910. 10.1007/s00122-008-0948-3 19130031

[B40] Li H. B. Vaillancourt R. Mendham N. Zhou M. X. (2008). Comparative mapping of quantitative trait loci associated with waterlogging tolerance in barley (*Hordeum vulgare* L.). *BMC Genomics* 9:401. 10.1186/1471-2164-9-401 18752688 PMC2533678

[B41] Li J. Huang X. Heinrichs F. Ganal M. Röder M. (2005). Analysis of QTLs for yield, yield components, and malting quality in a BC3-DH population of spring barley. *Theor. Appl. Genet.* 110 356–363. 10.1007/s00122-004-1847-x 15549229

[B42] Liu X. Fan Y. Mak M. Babla M. Holford P. Wang F. (2017). QTLs for stomatal and photosynthetic traits related to salinity tolerance in barley. *BMC Genomics* 18:9. 10.1186/s12864-016-3380-0 28049416 PMC5210286

[B43] Lumba S. Toh S. Handfield L. F. Swan M. Liu R. Youn J. Y. (2014). A mesoscale abscisic acid hormone interactome reveals a dynamic signaling landscape in *Arabidopsis*. *Dev. Cell* 29 360–372. 10.1016/j.devcel.2014.04.004 24823379

[B44] Ma Y. Shabala S. Li C. Liu C. Zhang W. Zhou M. (2015). Quantitative trait loci for salinity tolerance identified under drained and waterlogged conditions and their association with flowering time in barley (*Hordeum vulgare*. L). *PLOS ONE* 10:e0134822. 10.1371/journal.pone.0134822 26247774 PMC4527667

[B45] Malik A. I. Colmer T. D. Lambers H. Schortemeyer M. (2001). Changes in physiological and morphological traits of roots and shoots of wheat in response to different depths of waterlogging. *Funct. Plant Biol.* 28 1121–1131. 10.1071/PP01089

[B46] Mano Y. Komatsuda T. (2002). Identification of QTLs controlling tissue-culture traits in barley (*Hordeum vulgare* L.). *Theor. Appl. Genet.* 105 708–715. 10.1007/s00122-002-0992-3 12582484

[B47] Mano Y. Omori F. (2009). High-density linkage map around the root aerenchyma locus Qaer1.06 in the backcross populations of maize Mi29 × teosinte “*Zea nicaraguensis*”. *Breed. Sci.* 59 427–433. 10.1270/jsbbs.59.427

[B48] Mano Y. Takeda K. (1997). Mapping quantitative trait loci for salt tolerance at germination and the seedling stage in barley (*Hordeum vulgare* L.). *Euphytica* 94 263–272. 10.1023/A:1002968207362

[B49] Massalski C. Bloch J. Zebisch M. Steinebrunner I. (2015). The biochemical properties of the Arabidopsis ecto-nucleoside triphosphate diphosphohydrolase AtAPY1 contradict a direct role in purinergic signaling. *PLOS ONE* 10:e0115832. 10.1371/journal.pone.0115832 25822168 PMC4379058

[B50] Munns R. James R. A. Läuchli A. (2006). Approaches to increasing the salt tolerance of wheat and other cereals. *J. Exp. Bot.* 57 1025–1043. 10.1093/jxb/erj100 16510517

[B51] Newman I. (2001). Ion transport in roots: measurement of fluxes using ion-selective microelectrodes to characterize transporter function. *Plant Cell Environ.* 24 1–14. 10.1046/j.1365-3040.2001.00661.x 11762438

[B52] Palmgren M. G. Nissen P. (2011). P-type ATPases. *Annu. Rev. Biophys.* 40 243–266. 10.1146/annurev.biophys.093008.131331 21351879

[B53] Pang J. Zhou M. Mendham N. Shabala S. (2004). Growth and physiological responses of six barley genotypes to waterlogging and subsequent recovery. *Crop Pasture Sci.* 55 895–906. 10.1071/AR03097

[B54] Pang J. Y. Cuin T. Shabala L. Zhou M. X. Mendham N. Shabala S. (2007). Effect of secondary metabolites associated with anaerobic soil conditions on ion fluxes and electrophysiology in barley roots. *Plant Physiol.* 145 266–276. 10.1104/pp.107.102624 17660351 PMC1976565

[B55] Qiu L. Wu D. Ali S. Cai S. Dai F. Jin X. (2011). Evaluation of salinity tolerance and analysis of allelic function of *HvHKT1* and *HvHKT2* in Tibetan wild barley. *TAG Theor. Appl. Genet.* 122 695–703. 10.1007/s00122-010-1479-2 20981400

[B56] Rivandi J. Miyazaki J. Hrmova M. Pallotta M. Tester M. Collins N. (2010). A *SOS3* homologue maps to *HvNax4*, a barley locus controlling an environmentally sensitive Na^+^ exclusion trait. *J. Exp. Bot.* 62 1201–1216. 10.1093/jxb/erq346 21047983 PMC3022402

[B57] Robertson D. Zhang H. Palta J. A. Colmer T. Turner N. C. (2009). Waterlogging affects the growth, development of tillers, and yield of wheat through a severe, but transient. N deficiency. *Crop Pasture Sci.* 60 578–586. 10.1071/CP08440

[B58] Setter T. Waters I. (2003). Review of prospects for germplasm improvement for waterlogging tolerance in wheat, barley and oats. *Plant Soil* 253 1–34. 10.1023/A:1024573305997

[B59] Shabala S. (2011). Physiological and cellular aspects of phytotoxicity tolerance in plants: the role of membrane transporters and implications for crop breeding for waterlogging tolerance. *New Phytol.* 190 289–298. 10.1111/j.1469-8137.2010.03575.x 21563365

[B60] Shabala S. Bose J. Fuglsang A. T. Pottosin I. (2016). On a quest for stress tolerance genes: membrane transporters in sensing and adapting to hostile soils. *J. Exp. Bot.* 67 1015–1031. 10.1093/jxb/erv465 26507891

[B61] Shabala S. Pottosin I. (2014). Regulation of potassium transport in plants under hostile conditions: implications for abiotic and biotic stress tolerance. *Physiol. Plant.* 151 257–279. 10.1111/ppl.12165 24506225

[B62] Shavrukov Y. Gupta N. K. Miyazaki J. Baho M. N. Chalmers K. J. Tester M. (2010). *HvNax3*—a locus controlling shoot sodium exclusion derived from wild barley (*Hordeum vulgare* ssp. *spontaneum*). *Funct. Integr. Genomic.* 10 277–291. 10.1007/s10142-009-0153-8 20076983

[B63] Slavich P. Read B. Cullis B. R. (1990). Yield response of barley germplasm to field variation in salinity quantified using the EM-38. *Aust. J. Exp. Agric.* 30 551–556. 10.1071/EA9900551

[B64] Smethurst C. F. Garnett T. Shabala S. (2005). Nutritional and chlorophyll fluorescence responses of lucerne (*Medicago sativa*) to waterlogging and subsequent recovery. *Plant Soil* 270 31–45. 10.1007/s11104-004-1082-x

[B65] Takeda K. Fukuyama T. (1986). Variation and geographical distribution of varieties for flooding tolerance in barley seeds. *Barley Genet. Newsl.* 16 28–29.

[B66] Teakle N. L. Bazihizina N. Shabala S. Colmer T. D. Barrett-Lennard E. G. Rodrigo-Moreno A. (2013). Differential tolerance to combined salinity and O_2_ deficiency in the halophytic grasses *Puccinellia ciliata* and *Thinopyrum ponticum*: the importance of K^+^ retention in roots. *Environ. Exp. Bot.* 87 69–78. 10.1016/j.envexpbot.2012.09.006

[B67] Tounsi S. Ben Amar S. Masmoudi K. Sentenac H. Brini F. Véry A.-A. (2016). Characterization of two HKT1; 4 transporters from *Triticum monococcum* to elucidate the determinants of the wheat salt tolerance *Nax1* QTL. *Plant Cell Physiol.* 57 2047–2057. 10.1093/pcp/pcw123 27440547

[B68] Ullrich W. R. (2002). “Salinity and nitrogen nutrition,” in *Salinity: environment–plants–molecules*, eds Läuchli A. Lüttge U. (Dordrecht: Kluwer), 229–248.

[B69] Van Ooijen J. W. (2006). *Joinmap 4.0 Software for the Calculation of Genetic Linkage Maps In Experimental Populations.* Wageningen: Kyazma B. V.

[B70] Van Ooijen J. W. (2009). *MapQTL 6.0 Software for the Mapping of Quantitative Trait Loci in Experimental Populations of Dihaploid Species.* Wageningen: Kyazma B. V.

[B71] Vitart V. Baxter I. Doerner P. Harper J. F. (2001). Evidence for a role in growth and salt resistance of a plasma membrane H^+^-ATPase in the root endodermis. *Plant J.* 27 191–201. 10.1046/j.1365-313x.2001.01081.x 11532165

[B72] Voorrips R. E. (2002). MapChart: Software for the graphical presentation of linkage maps and QTLs. *J. Hered.* 93 77–78. 10.1093/jhered/93.1.77 12011185

[B73] Wang J. Yang J. McNeil D. L. Zhou M. (2010). Identification and molecular mapping of a dwarfing gene in barley (*Hordeum vulgare* L.) and its correlation with other agronomic traits. *Euphytica* 175 331–342. 10.1007/s10681-010-0175-2

[B74] Wang J. Yang J. Zhang Q. Zhu J. Jia Q. Hua W. (2015). Mapping a major QTL for malt extract of barley from a cross between TX9425 × Naso Nijo. *Theor. Appl. Genet.* 128 943–952. 10.1007/s00122-015-2481-5 25773294

[B75] Wang M. Zheng Q. Shen Q. Guo S. (2013). The critical role of potassium in plant stress response. *Int. J. Mol. Sci.* 14 7370–7390. 10.3390/ijms14047370 23549270 PMC3645691

[B76] Xu R. Wang J. Li C. Johnson P. Lu C. Zhou M. (2012). A single locus is responsible for salinity tolerance in a Chinese landrace barley (*Hordeum vulgare* L.). *PLOS ONE* 7:e43079. 10.1371/journal.pone.0043079 22916210 PMC3423432

[B77] Xue D. Huang Y. Zhang X. Wei K. Westcott S. Li C. (2009). Identification of QTLs associated with salinity tolerance at late growth stage in barley. *Euphytica* 169 187–196. 10.1007/s10681-009-9919-2

[B78] Xue D. W. Zhou M. X. Zhang X. Q. Chen S. Wei K. Zeng F. R. (2010). Identification of QTLs for yield and yield components of barley under different growth conditions. *J. Zhejiang Univ. Sci. B* 11 169–176. 10.1631/jzus.B0900332 20205303 PMC2833401

[B79] Yamaguchi T. Blumwald E. (2005). Developing salt-tolerant crop plants: challenges and opportunities. *Trends Plant Sci.* 10 615–620. 10.1016/j.tplants.2005.10.002 16280254

[B80] Zaidi P. H. Rashid Z. Vinayan M. T. Almeida G. D. Phagna R. K. Babu R. (2015). QTL mapping of agronomic waterlogging tolerance using recombinant inbred lines derived from tropical maize (*Zea mays* L) germplasm. *PLOS ONE* 10:e0124350. 10.1371/journal.pone.0124350 25884393 PMC4401703

[B81] Zeng F. Konnerup D. Shabala L. Zhou M. Colmer T. D. Zhang G. (2014). Linking oxygen availability with membrane potential maintenance and K^+^ retention of barley roots: implications for waterlogging stress tolerance. *Plant Cell Environ.* 37 2325–2338. 10.1111/pce.12422 25132404

[B82] Zhang X. Shabala S. Koutoulis A. Shabala L. Johnson P. Hayes D. (2015). Waterlogging tolerance in barley is associated with faster aerenchyma formation in adventitious roots. *Plant Soil* 394 355–372. 10.1007/s11104-015-2536-z

[B83] Zhang X. Shabala S. Koutoulis A. Shabala L. Zhou M. (2017). Meta-analysis of major QTL for abiotic stress tolerance in barley and implications for barley breeding. *Planta* 245 283–295. 10.1007/s00425-016-2605-4 27730410

[B84] Zhang X. Tang B. Yu F. Li L. Wang M. Xue Y. (2013). Identification of major QTL for waterlogging tolerance using genome-wide association and linkage mapping of maize seedlings. *Plant Mol. Biol. Rep.* 31 594–606. 10.1007/s11105-012-0526-3

[B85] Zhang X. C. Zhou G. F. Shabala S. Koutoulis A. Shabala L. Johnson P. (2016). Identification of aerenchyma formation-related QTL in barley that can be effective in breeding for waterlogging tolerance. *Theor. App. Genet.* 129 1167–1177. 10.1007/s00122-016-2693-3 26908252

[B86] Zhou M. (2010). “Improvement of plant waterlogging tolerance,” in *Waterlogging Signalling and Tolerance in Plants*, eds Mancuso S. Shabala S. (Berlin: Springer), 267–285.

[B87] Zhou M. Johnson P. Zhou G. Li C. Lance R. (2012). Quantitative trait loci for waterlogging tolerance in a barley cross of Franklin × YuYaoXiangTian Erleng and the relationship between waterlogging and salinity tolerance. *Crop Sci.* 52 2082–2088. 10.2135/cropsci2012.01.0008

[B88] Zhou M. Li H. Mendham N. (2007). Combining ability of waterlogging tolerance in barley. *Crop Sci.* 47 278–284. 10.2135/cropsci2006.02.0065

[B89] Zhou M. X. (2011). Accurate phenotyping reveals better QTL for waterlogging tolerance in barley. *Plant Breed.* 130 203–208. 10.1111/j.1439-0523.2010.01792.x

