# Supplementary material for: Cell-Based Phenotyping Reveals QTL for Membrane Potential Maintenance Associated with Hypoxia and Salinity Stress Tolerance in Barley
Source: Front Plant Sci. 2017 Nov 16;8:1941. doi: 10.3389/fpls.2017.01941 (PMC5696338; doi:10.3389/fpls.2017.01941)
Supplement: Supplementary file 1 [file Table_1.DOCX]

| Source of Variation | SS | df | MS | F | P-value | F crit |
| --- | --- | --- | --- | --- | --- | --- |
| Between Groups | 376649.7 | 149 | 2527.85 | 40.23671 | 4.8E-192 | 1.23783 |
| Within Groups | 28271.02 | 450 | 62.82448 |  |  |  |
| Total | 404920.7 | 599 |  |  |  |  |

**Supplementary Table S1.** Anova of membrane potential values under waterlogging (hypoxia) stress.
